# Efficacy of interpersonal psychotherapy in mainland China: a systematic review and meta-analysis

**DOI:** 10.3389/fpsyt.2023.1160081

**Published:** 2023-07-12

**Authors:** Luhan Tang, Fangzhong Xu, Ge Yu, Chong Li, Sijin Wen, Wanhong Zheng

**Affiliations:** ^1^Department of Clinical Psychology, Tong De Hospital of Zhejiang Province, Hangzhou, Zhejiang, China; ^2^Department of Mathematics, West Virginia University, Morgantown, WV, United States; ^3^Department of Epidemiology and Biostatistics, West Virginia University, Morgantown, WV, United States; ^4^Department of Behavioral Medicine and Psychiatry, West Virginia University, Morgantown, WV, United States

**Keywords:** IPT, interpersonal psychotherapy, China, psychotherapy, therapy

## Abstract

**Objective:**

Interpersonal Psychotherapy (IPT) is an evidence-based therapy. There have been increasing demand and training opportunities of IPT in China. Reviewing current evidence on its use in Chinese patients can help us understand the applicability of IPT in China and identify knowledge gaps to encourage and better future research in this field.

**Method:**

We did a comprehensive search of three major electronic databases: PubMed (English), Chinese National Knowledge Infrastructure (CNKI) and WanFang Data (Chinese). We examined overall study design, outcome measures, data analyses and other parameters. We only selected articles of Randomized Clinical Trials (RCT) for this review. All study findings were grouped and summarized per psychiatric diagnoses. The meta-analysis and forest plots were performed whereas studies could be combined.

**Results:**

After a full text review of 132 articles, 40 were selected for the final review. Comparing with control groups, evidences supported the efficacy of IPT in Chinese patients with Major Depressive Disorder (MDD), Postpartum depression, Generalized Anxiety Disorder (GAD), Social Anxiety Disorder, Post Stress Traumatic Disorder (PTSD), and Post-psychotic Depression. It was also beneficial to college students and Chinese first-time mothers. Meta-analysis using a random-effects model consistently yielded significant score differences between the IPT and control groups (*p* < 0.0001) on MDD.

**Conclusion:**

This systematic review has identified the current best evidence for IPT efficacy in Chinese population. The findings support IPT as an effective treatment in Chinese with certain psychiatric conditions, consistent with those from many other studies throughout the world.

## 1. Introduction

IPT is a time-limited, problem area focused, and evidence-based therapy to treat different psychiatric conditions (1). Originally developed to treat MDD, IPT has been used effectively to help patients with various diagnoses including bipolar disorder, eating disorder, perinatal depression, etc. (2).

IPT is a well-studied psychotherapy. Throughout the past five decades, more than 250 randomized controlled trials of IPT have been published by various research groups around the world (3). With or without adaptation, IPT has demonstrated a clear efficacy as both an acute and maintenance treatment option for major depression (4), bipolar disorder (5), PTSD (6), anxiety disorders (7) and eating disorders (8) for the target populations across lifespan. It has been proved to be superior to placebo, similar to medications and mostly more efficacious when combined with medications in treating depression (9–11). In anxiety disorder, IPT has shown same effect compared to CBT, and greater benefits compared to treatment as usual controls (7). In addition, a recent systematic review concluded IPT was non-inferior to CBT in treating patients with anorexia nervosa, and had same long-term efficacy in patients with bulimia nervosa (8). While it is intuitively obvious psychotherapy generally gives less side effects comparing with pharmacologic treatment, there was no report on whether IPT may adversely impact on patient’s psychological or physical condition. On the other hand, some studies have concluded that IPT is one of the most const-effective interventions in certain patient populations (12). Because of all these reasons, IPT has been included in different treatment guidelines of many countries such as Australia, Canada, United Kingdom, and United States (13). In 2015, World Health Organization (WHO) updated the Mental Health Gap Action Programme (mhGAP) guidelines and recommended IPT as one of major psychological interventions for adults, older and adolescents with depressive disorder (14). It is also listed as the first-line psychological treatment for acute MDD in the Canadian Network for Mood and Anxiety Treatments (CANMAT) guidelines (15).

China, as one of the largest countries in the world, has a current population over 1.4 billion (16). Estimated by World Health Organization (WHO), about 54 million people in China have depression and 41 million suffer from anxiety disorders (17). Over the past three decades, Chinese government has made significant efforts to expand mental health resources and increase access to treatment. Same as pharmacologic management, psychotherapy has nowadays become recognized as a scientific and effective treatment method, and has been gradually adopted as a main treatment modality in mental health care (18).

Despite good evidence for efficacy, IPT has not been commonly practiced in China. As many practitioners in China are focused on psychoanalysis and CBT as the mainstream psychotherapy options, IPT remains as an unfamiliar treatment option for majority of Chinese therapists. Given the fact that there has been an increasing demand in evidence-based psychotherapy, and rapidly growing training opportunities of IPT in China, it is important to review the up-to-date IPT related clinical studies that have been conducted on Chinese participants. As our initial online database search did not return enough number of English articles targeting Chinese subjects, we were curious to know whether many were published in Chinese. This article aims to summarize the findings of the existing studies published in the medical literature (in both Chinese and English) on the efficacy of IPT in mainland China. To our knowledge, there has been no previous systematic review undertaken on this topic. Analysis of current studies can surely help us understand the applicability of IPT in Chinese population and identify knowledge gaps to encourage and better future research in this field.

## 2. Materials and methods

### 2.1. Inclusion and exclusion criteria

This systematic review focused on reports and studies of IPT in mainland China so the main target population is Chinese. We defined IPT as the structured psychotherapy that was conceptualized by Klerman, Weissman, and colleagues. We also included its derivative adaptations such as Group IPT, Brief IPT (IPT-B) and IPT-oriented Education.

The inclusion criteria of this review were:

- Studies were published in English or Chinese
- Studies were original RCTs
- Studies of IPT. A psychotherapy was considered IPT if it was a derivative or adaptation of the initial operationalized IPT (1), with similar techniques and interpersonal focus.
- Target participants were Chinese, and the intervention was conducted in mainland China

### 2.2. Search strategy

As the scope of this review is limited to IPT studies in mainland China, we predicted that some articles could be published in Chinese instead of English. Therefore, the electronic databases we searched included both languages: PubMed (in English), Chinese National Knowledge Infrastructure (CNKI) and WanFang Data (in Chinese). We used database-specific combinations of the following terms or words: interpersonal psychotherapy, IPT, interpersonal therapy, China, mainland China and Chinese. There was no time limit, so all articles published before September 2022 in all three databases were included.

### 2.3. Study selection and quality assessment

Study selection and quality assessment were conducted by three of the authors. All titles and abstracts of the selected studies were first independently screened by two reviewers (LHT and GY). Then the three reviewers independently screened the studies for eligibility according the inclusion and exclusion criteria by reading the full text carefully. Any disagreements were resolved by discussions among the three reviewers (LHT, FZX and GY). We examined overall study design, selection bias, confounders, data collection methods, outcome measures and data analyses. Much attention was paid to the consistency and clarity of reporting. To assure the quality, we only selected RCT studies for final analysis. Case reports, commentaries, non-journal articles, qualitative studies, open trials and studies with inconsistent data were excluded. Meta-analyses were conducted when appropriate. Whereas studies could not be combined, summaries were provided.

### 2.4. Meta-analysis

The meta-analysis and forest plots were performed using the meta-analysis package “meta” (19, 20) and statistical software R (version 3.6.3, R Foundation, Vienna, Austria). The combined mean difference (MD) between intervention and control groups with its 95% confidence interval was estimated based on a random-effects model, taking into account between and within variation from different studies. Heterogeneity was examined based on τ2 statistic and I2 statistic from either random-effects model (if significant) or fixed-effects model (if not significant) (20).

## 3. Results

### 3.1. Study design and sample size

**Figure 1:**
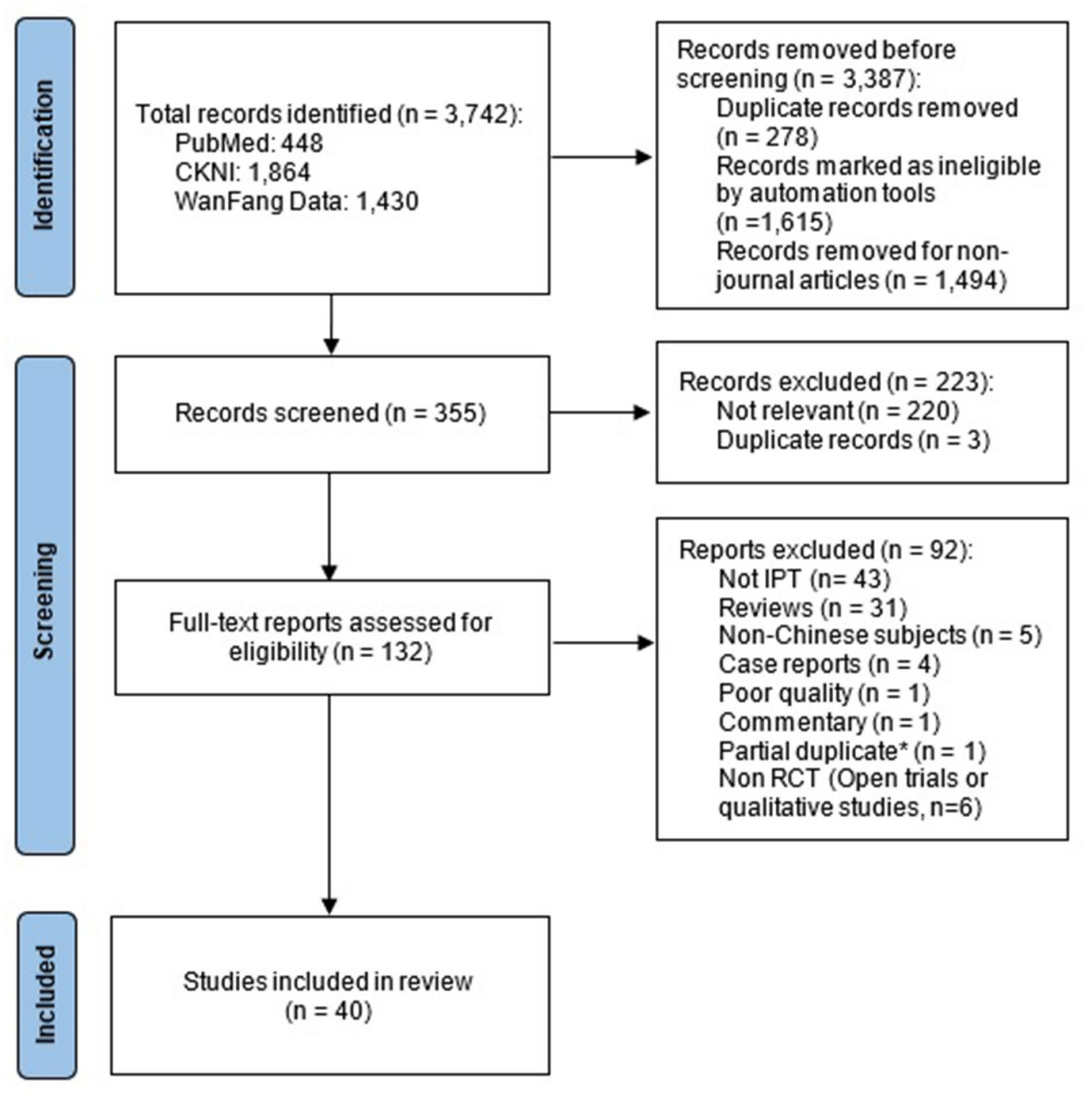
Prisma flow diagram of study selection process * one study was published with additional CBT comparison so we only included the new publication.

A total of 3,742 articles were initially retrieved from the databases. After excluding all duplicates and removing non-journal articles, application of inclusion criteria from the titles generated 355 articles and finally 132 articles were selected for full text review. Here, 40 studies were included in the final review. The PRISMA flow chart (21) is presented in Figure 1. Included in our analysis are 40 RCTs. The number of subjects in the studies varied from 21 to 176 with majority (57.5%) involved 80 or more participants. Table 1 provides a summary of all included studies.

**Table 1:** Summary of included studies (*n* = 40).

References	Language	Sample characteristics	Diagnostic criteria	Study design	Intervention	Outcome measurement	Main outcomes	Statistical significance
	MDD (general population)							
Ye and Li (22)	Chinese	Age:18–60, *n* = 60	DMS-IV	RCT	Group IPT (12 weekly, 90 min) + antidepressant vs. antidepressant only	HAMD, SDSS	The symptom alleviation and social function improvement were more significant in IPT + medicine group.	*p* < 0.01
Ying et al. (23)	Chinese	Age:36.2 ± 13, *n* = 82	CCMD-3	RCT	Individual IPT (12 weekly, 50–60 min) + SSRI vs. SSRI only	HAMD, Recurrence rate per CCI-CI after 6 months	The effective rates were 83.3% in the IPT and 60.0% in the control group. Recurrence rate of IPT group (7.1%) was also better than control group (22.5%).	*p* < 0.05
Qiao and Niu (24)	Chinese	Age:18–50, *n* = 40	DSM-IV	RCT	Group IPT (10 weekly, 90 min) + medicine vs. medicine only	HAMD, SDSS	The symptom alleviation and social function improvement were more significant in IPT + medicine group.	*p* < 0.01
Zhu et al. (25)	Chinese	Age:37.63 ± 6.78, *n* = 58	DSM-IV	RCT	Group IPT (12 weekly, 90 min) + medicine vs. medicine only	HAMD, SDSS	The symptom alleviation and social function improvement were more significant in IPT + medicine group.	*p* < 0.01
Kang et al. (26)	Chinese	TRD, Age:34.3 ± 10.5, *n* = 100,	ICD-10	RCT	Individual IPT (12 weekly, 50-60 min) + escitalopram + Olanzapine vs. medicine only	HAMD, HAMA, SF-36	IPT combined with medications had better treatment effect (90.0%) than control group (72.0%). IPT improved the quality of life.	*p* < 0.05
Kong (27)	Chinese	Age:20–50, *n* = 80	ICD -10	RCT	Group IPT (10 weekly, 100 min) + medicine vs. medicine only	HAMD, SDSS	Significant reduction in depression and improvement in social function of IPT group comparing with medicine only group.	*p* < 0.01
Wang and Zhang (28)	Chinese	Age: 38.6 ± 5.2, *n* = 66	DSM-IV	RCT	Group IPT (12 weekly, 90 min) + medicine vs. medicine only	HAMD, SDSS	Significant reduction in depression and improvement in social function of IPT group comparing with medicine only group.	*p* < 0.05
Ren and Lei (29)	Chinese	Age:23–82, *n* = 86	Clinically documented diagnoses by psychiatrists	RCT	Individual IPT + medicine (16 weekly, 50 min) vs. medicine only	SDSS, SSRS	The social functioning and social support more significantly improved in IPT group.	*p* < 0.001
Wen et al. (30)	Chinese	Age:18–50, *n* = 57	ICD-10	RCT	Group IPT (6 weeks, 2 sessions per week, each session 120 min) + SSRIs vs. TNC + SSRIs	HAMD, SAD, SDS	Both groups demonstrated significant improvement in depression but IPT group had more improvement in social anxiety and social avoidance.	*p* < 0.05
Xu et al. (31)	Chinese	Age:18–60, *n* = 60	DSM-5	RCT	Individual IPT (16 weeks, 45 min) + medicine vs. medicine only	HAMD, SDSS, SSRS	Significant reduction in depression and improvement in social function of IPT group comparing with medicine only group.	*p* < 0.05
	MDD (geriatrics)							
Sun et al. (32)	Chinese	Age: 65.3 ± 3.9, *n* = 84	ICD-10	RCT	Group IPT (12 weekly, 90 min) + escitalopram vs. escitalopram only	HAMD, SDSS GQOLI-74	IPT group had more improvement in depressive symptoms, social function and quality of life in depressed geriatric patients.	*p* < 0.05
Wang and Li (33)	Chinese	Age: 65–83, *n* = 98	Clinically documented diagnoses by psychiatrists	RCT	Group IPT (12 weekly, 90 min) + paroxetine vs. paroxetine only	HAMD, SDSS, EPDS	IPT group had more improvement in depressive symptoms, social function and quality of life in depressed geriatric patients.	*p* < 0.05
	MDD (adolescents)							
Sun (34)	Chinese	Children of economic migrants Age: 13.05 ± 0.81, *n* = 21	BDI ≥ 14	RCT	Group IPT-B (6 weekly, 90 min) vs. no treatment	BDI	IPT group had better improvement in BDI than control group.	*p* < 0.001
	Postpartum depression							
Rao et al. (35)	Chinese	Age: 27.5 ± 13, *n* = 110	DSM-IV	RCT	Group IPT (8 weekly, 90 min) vs. CBT vs. TNC	EPDS, HAMD, DAS, IIP	Both CBT and IPT had better clinical outcomes than the control group. CBT group maintained better effect than IPT at 6-month follow-up.	*p* < 0.05
Liu (36)	Chinese	Age: 19–35, *n* = 120	Not mentioned	RCT	IPT (8 weeks, details not provided) vs. TNC	EPDS	Significant reduction in EPDS scores at week 4 and 8 in IPT group in comparison to TNC.	*p* < 0.01
Shi and Li (37)	Chinese	Primipara women Age: 23–37, *n* = 108	HAMD≥17	RCT	Group IPT (12 weekly, 60 min) vs. TNC	HAMD, SDSS, Self-report of parenting competence for neonates, breast milk production	IPT group showed better reduction in HAMD, SDSS scores, higher scores of self-report of parenting competence for neonates and more breast milk production	*p* < 0.05
Jin et al. (38)	Chinese	Age: 20–35, *n* = 60	DSM-IV	RCT	Individual IPT (12 weekly, 50 min) vs. CBT	EPDS, SSRS	Both IPT and CBT had treatment effect on postpartum depression. IPT group had a better effect on improving social support.	*p* < 0.01
Wang (39)	Chinese	Age: 29.7 ± 1.8, *n* = 86	Clinically documented diagnoses by psychiatrists	RCT	Group IPT (8 weekly, 90 min) vs. TNC	EPDS, IIP, SDSS	IPT group had better improvements in all 3 outcome measurements.	*p* < 0.05
Lin (40)	Chinese	Age: 28.11 ± 2.42, *n* = 80	Not mentioned	RCT	Individual IPT (12 weekly, 50 min) vs. TNC	HAMD, HAMA	IPT group showed better reduction in HAMD, HAMA scores and postpartum depression incidence rate.	*p* < 0.05
	MDD (patients with medical conditions)							
Du (41)	Chinese	Diabetic patients with depression Age: 59.78 ± 6.57, *n* = 62	DSM-IV	RCT	Group IPT + antidepressant (9 weekly, 90 min) vs. antidepressant only	HAMD, FBG, 2hPBG, HbA1c	Both IPT group and control group demonstrated reduced depressive symptoms. IPT group also had better blood sugar control after treatment.	*p* < 0.05
Hu (42)	Chinese	Diabetic Patients with depression Age: 62.7 ± 8.7, *n* = 120	HAMD > 17	RCT	Group IPT + antidepressant (8 weekly, 90 min) vs. antidepressant only	HAMD, FBG, 2hPBG, HbA1c	Both IPT group and control group demonstrated effectiveness in reducing depressive symptoms and blood sugar control. IPT group had higher rate of responses.	*p* < 0.05
Jiang and Li (43)	Chinese	MHD depression. Age not reported, *n* = 44	DMS-IV	RCT	Group IPT (14 weekly, 60–90 min) vs. TAU	SDS	Comparing with TAU, IPT group had a significant reduction in SDS score after treatment.	*p* < 0.01
	GAD							
Huang et al. (44)	Chinese	Age:35.56 ± 11.51, *n* = 90	CCMD-3	RCT	Individual IPT (12 weekly, 50 min) vs. CBT	HAMA, HAMD	Both IPT and CBT were effective. CBT group had better effect on sleep disturbance	*p* < 0.05
Liang (45)	Chinese	Age: 35.6 ± 11.5, *n* = 80	Clinically documented diagnoses by psychiatrists	RCT	Individual IPT (12 weekly, 50 min) vs. CBT	HAMD, HAMA, QOL	Both IPT and CBT were effective. CBT group had better effect on improving quality of life.	*p* < 0.05
Li (46)	Chinese	Age: 36.26 ± 3.05, *n* = 84	CCMD-3	RCT	Individual IPT (12 weekly, 50 min) vs. CBT	HAMD, HAMA Quality of Life	Both IPT and CBT had treatment effect on GAD. CBT group had a better effect on improving quality of life.	*p* < 0.05
	Social anxiety disorder							
Huang and Liu (47)	Chinese	Age:20 ± 2, *n* = 45	DSM-IV	RCT	Group IPT (8 weekly， 120 min) vs. CBT vs. Waiting list	IAS, SADS	Both IPT and CBT had better treatment effect on social anxiety than the control group.	*p* < 0.05
Lin et al. (48)	Chinese	Age: 21.25 ± 4.4, *n* = 43	DSM-IV	RCT	Group IPT (12 weekly, 120 min) vs. no treatment	SADS, RESE questionnaire	IPT group showed improvement in perceived self-efficacy in expressing positive affect (POS) and reduction in social avoidance and distress.	*p* < 0.05
	PTSD or patients with childhood trauma							
Jiang et al. (49)	English	Earthquake survivors Age: 24.79 ± 11.66, *n* = 49	DSM-IV	RCT	Individual IPT (12 weekly 60 min) vs. TAU	CAPS, SCID, BDI-II, GSE, CTS, STAXI, SAS, QLI	A significantly greater reduction of PTSD and MDD diagnoses was found in the IPT group (51.9, 30.1%, respectively) versus the TAU (3.4, 3.4%, respectively). Treatment gains were maintained at 6 months for the IPT group.	*p* < 0.01
Li et al. (50)	Chinese	College students with anxiety and childhood abuse Age: 18–21, *n* = 48	DSM-IV	RCT	Individual IPT (6 weeks，2 sessions/week, 30–40 min) vs. supportive psychotherapy	SAS, GAF, CD-RISC, SRFIS, DSC	IPT could alleviate the anxiety and depression symptoms and improve psychosocial functionality in college students with anxiety and history of childhood abuse.	*p* < 0.05
	Post-psychotic depression							
Zhou et al. (51)	Chinese	Age: 18–55, *n* = 82	CCMD-3	RCT	Group IPT (10 weekly, 60–90 min) + medicine vs. medicine only	SCL-90, SDSS	IPT had better treatment effect on stable psychotic patients’ clinical symptoms and social functionality than control group.	*p* < 0.05
Lu and Chen (52)	Chinese	Age: 34 ± 12, *n* = 82	ICD-10	RCT	Individual IPT (12 weekly) + medicine vs. medicine only	HAMD	IPT had better clinical efficacy in depression treatment than control group. There was also a difference in medication compliance between the two groups.	*p* < 0.05
Zhou (53)	Chinese	Age: 34.1 ± 5.91, *n* = 68	ICD-10	RCT	Individual IPT (12 weekly) + medicine vs. medicine only	HAMD	Both IPT and control group showed improvement in depression after 1 month. IPT group had better continuous improvements than control after at 2 and 3-month follow-ups.	*p* < 0.001
Kang (54)	Chinese	Age: 34.76 ± 3.98, *n* = 92	CCMD-3	RCT	Individual IPT (6 sessions, 3 CBT + 3 IPT) + paroxetine vs. paroxetine only	HAMD, SES, PANSS, WHO-DAS II, medication compliance	IPT combined with CBT could improve depression, self-esteem, social functioning and medication compliance in patients with post-schizophrenia depression.	*p* < 0.05
	Special population and other diagnoses							
Li et al. (55)	English	College students with aggression Age: 18–19, *n* = 60	WFSBP guidelines	RCT	Group IPT (16 weekly, 60 min) vs. no treatment	CC-BPAQ, the Social Support Scale for University Students	IPT was effective in treating aggression and improving social support level for college students.	*p* < 0.05
Li et al. (56)	Chinese	College students with aggression Age 18–19, *n* = 89	CC-BPAQ > mean + SD	RCT	Group IPT (16 weekly, 60 min) vs. Group CBT vs. no treatment	CC-BPAQ	Both IPT group and CBT group had reduced the aggressiveness level after treatment, but IPT was superior in terms of CC-BPAQ scores.	*p* < 0.01
Guang and Li (57)	Chinese	Somatoform disorder patients Age: 41.5 ± 9.2, *n* = 81	ICD-10	RCT	Individual IPT (12 weeks, 50–60 min, 2 sessions/week) + duloxetine vs. CBT + duloxetine vs. duloxetine only	HAMD, HAMA	Both IPT (88.89%) and CBT (85.19%) had better therapeutic effect on depression and anxiety than control group (59.26%).	*p* < 0.05
Chen et al. (58)	Chinese	Bipolar disorder patients Age: 42.3 ± 10.3, *n* = 150	Clinically diagnosed, HAMD≥17, YMRS<20	RCT	Group IPT (8 weekly, 60 min) + medicine vs. medicine only	HAMD, YMRS, WHOQOL_BREF	IPT is effective for bipolar depression and could improve the quality of life.	*p* < 0.05
	Others (IPT-oriented education)							
Gao et al. (59)	English	First-time childbearing women Age: ≤ 35, *n* = 175	Not mentioned	RCT	IPT-oriented childbirth education (2 sessions, 90 min, + 1 telephone follow-up) vs. Routine childbirth education	EPDS, GHQ, SWIRS	IPT group had significantly better psychological well-being, fewer depressive symptoms and better interpersonal relationships at 6 weeks postpartum as compared with control group.	*p* < 0.001
Gao et al. (60)	English	First-time childbearing women Age: ≤35, *n* = 175	Not mentioned	RCT	IPT-oriented childbirth education (2 sessions, 90 min, + 1 telephone follow-up) vs. Routine childbirth education	PSSS, PSOC, EPDS, GHQ	Women in IPT-oriented education had better social support, better maternal role competence and less depressive symptoms than those in control group.	*p* < 0.01
Gao et al. (61)	English	First-time mothers Age: 28.49 ± 2.73 and 28.67 ± 2.91 (control), *n* = 176	Not mentioned	RCT	IPT-oriented childbirth education (2 sessions, 90 min, + 1 telephone follow-up) vs. Routine childbirth education	EPDS, PSSS, PSOC-E	Women receiving the IPT psychoeducation program had significantly fewer depressive symptoms, higher level of social support and better maternal role competence at 6 weeks postpartum.	*p* < 0.05

2hPBG, 2-h Postprandial Blood Sugar; BDI-II, Beck Depression Inventory; CAPS, Clinician Administered PTSD Scale; CBT, Cognitive Behavioral Therapy; CC-BPAQ, the Chinese Revision of the Buss-Perry Aggression Questionnaire; CCMD-3, Chinese Classification of Mental Disorders Version 3; CD-RISC, Connor-Davidson Resilience Scale; CES-D, Center for Epidemiological Survey, Depression Scale; CTS, Conflict Tactics Scale; DAS, Dyadic Adjustment Scale; DSC, Depression Symptom Checklist; DSM-IV, The Diagnostic and Statistical Manual of Mental Disorders, Fourth Edition; EPDS, Edinburgh Postnatal Depression Scale; EPDS-10, The Edinburgh Postnatal Depression Scale; ESRD, End-Stage Renal Disease; FBG, Fasting Blood Sugar; GAF, Global Assessment Function; GHQ, General Health Questionnaire; GQOLI-74, Generic Quality of Life Inventory-74; GSE, Generalized Self-Efficacy; HAMD, Hamilton Rating Scale for Depression – Chinese Version; HbA1c, Hemoglobin A1c; IAS, Interaction Anxiousness Scale; ICD-10, International Classification of Diseases, Tenth Revision; IIP, The Inventory of Interpersonal Problems; MDD, major Depressive Disorder; MHD, Maintenance hemodialysis; PANSS, The Positive and Negative Syndrome Scale; PSOC, Parenting Sense of Competence Scale; PSOC-E, Parenting Sense of Competence Scale – Efficacy subscale; PSOC-S, Parenting Sense of Competence Scale – Satisfaction subscale; PSSS, Perceived Social Support Scale; QLI, Quality of Life Index; QOL, Quality of Life; RESE, Regulatory Emotional Self-efficacy; SADS, Social Avoidance and Distress Scale; SAS, Self-rating Anxiety Scale; SAS, Social Adjustment Scale; SCID, Structured Clinical Interview for DSM-IV; SDS, The Zung Self-Rating Depression Scale; SDSS, Social Disability Screening Schedule; SES, Self Esteem Scale; SRFIS, Self-rating Feeling of Insecurity Scale; SSRI, Selective Serotonin Reuptake Inhibitor; SSRS, Social Support Rating Scale; SSRS, Social Support Scale; STAXI, State Trait Anger; SWIRS, Satisfaction with Interpersonal Relationships Scale; TAU, Treatment as Usual; TNC, Traditional Nursing Care; TRD, Treatment Resistant depression; WFSBP, World Federation of Societies of Biological Psychiatry; WHO-DAS II, The World Health Organization’s Disability Assessment Scale II.

### 3.2. Systematic review of IPT on Chinese patients with different psychiatric diagnoses

#### 3.2.1. MDD (general population)

Comparing with medication-only, combination of IPT with antidepressants is significantly more effective for depressive symptoms and social functioning improvement (22–31). Among the 10 studies we reviewed, 9 used HAMD, 7 included SDSS in outcome measurements. A meta-analysis was performed and the results are detailed in 3.3.

In summary, 4 studies compared individual IPT plus medicine with medicine only (23, 26 29, 31). Follow-up assessments consistently showed improvement in HAMA and HAMD scores at 8- and 12- weeks for both groups and the effect (score reduction) was superior in those who received IPT. One study (26) targeted TRD (treatment resistant depression) subjects (tried at least 2 antidepressants at optimal doses with clinically appropriate durations, current HAMD ≥18) showed IPT group had better quality of life as reflected by higher SF-36 scores after treatment. Two others (29, 30) included social functionality and social support as outcome measurements. Both found better improvement in SDSS and SSRS in IPT groups.

Six studies (22, 24, 25, 27, 28, 30) investigated the effectiveness of IPT-G for MDD patients. Antidepressant was also used by both intervention and control groups. The intervention groups received 10 to 12 sessions of 50- to 100-min group IPT. All studies were conducted on inpatient subjects and excluded those with active suicidal ideations. Again, the results of all studies supported statistically better HAMD improvement in those received both IPT-G and medications. Most also showed lower SDSS scores after treatment in both IPT-G and control groups, with better reduction in IPT-G patients (22, 24, 25, 27, 28).

#### 3.2.2. MDD (geriatrics)

Two studies conducted on Chinese geriatric patients found that 12 weekly IPT in combination with escitalopram or paroxetine showed more reductions in HAMDS and SDSS scores comparing with the medication-only group (*p* < 0.05) (32, 33). The sample sizes for the two studies were 84 and 98, both split evenly between IPT and control groups. One study (32) also checked GQOLI -74 and concluded that the total score and sub-scores of dimensions of social function and psychological function of the GQOLI -74 in the IPT group were significantly higher than those of the control group (*p* < 0.01), reflecting better overall self-reported wellbeing after IPT treatment. The results support that IPT and antidepressant together can help elder people improve depressive symptoms, social function, and quality of life.

#### 3.2.3. MDD (adolescents)

Unfortunately, only one RCT study targeting adolescent population was retrieved. This study investigated the effectiveness of group IPT-B (6 weekly 90-min sessions) in treating junior middle school adolescents whose parents were economic migrants (34). After screening 113 candidates who met the screening criteria, the research team recruited 21 subjects who scored moderate to severe depression on BDI. Among those, 11 were randomly selected for group IPT. The result showed only IPT group had significantly decreased BDI following the intervention and the treatment effect was maintained at 90-day follow-ups (*p* < 0.001).

#### 3.2.4. Postpartum depression

Six RCT studies showed promise of IPT for postpartum depression (35–40). One study compared 8 weekly 90-min IPT-G vs. 8 weekly 60-min CBT vs. traditional nursing care and concluded that comparing with traditional care, while both IPT-G and CBT had significantly better clinical outcomes in terms of EPDS, HAMD, DAS and IIP at 3 and 6 month follow ups, CBT group showed superiority in maintaining therapeutic effect in depression and dysfunctional attitudes (as measured by EPDS, HAMD and DAS) at 6-month follow-up (35). Three other studies (36, 37, 39) conferred the effectiveness of IPT-G in similar population comparing with TAU, and one found that those received IPT-G even achieved higher scores of self-report of parenting competence for neonates and more breast milk production (37). Furthermore, a recent study (38) compared individual IPT with CBT on 60 outpatients with PPD (30 for each group). Both groups received 12 weekly 50-min individual therapy sessions. The results showed reduced scores of EPDS and increased ratings in SSRS in both groups, indicating significant improvement in depression and social support. It also found that the total and sub scores of social support scale in IPT group were significantly higher than those in the CBT group (*p* < 0.05).

#### 3.2.5. MDD (patients with medical conditions)

Two studies investigated IPT-G use in diabetic patients with depression. The results showed that Group IPT plus antidepressant can effectively reduce depressive symptoms and better blood sugar control (*p* < 0.05) (41, 42). Comparing with antidepressant-only group, those who also received 8–9 weekly 90-min group IPT demonstrated better FBG, 2hPBG and HbA1c after treatment. For end stage renal disease (ESDR) patients on MHD, depression could significantly affect treatment compliance and overall life quality. One study in this population showed that IPT-G was effective in alleviating affective, cognitive, behavioral, and somatic symptoms of depression (*p* < 0.01) (43).

#### 3.2.6. GAD and social anxiety disorder

Three RCT studies compared IPT with CBT in treating GAD. Huang et al. studied 90 GAD patients who all scored ≥14 on HAMA at the time of recruitment (44). Thirty-one (31/45, complete rate 68.9%) completed 12 weekly 50-min IPT, and 33 (33/45, complete rate 73.3%) finished 12 weekly 50-min CBT. Both groups demonstrated significant reduction in HAMA total scores and a t-test showed no difference between the two groups in terms of reductions in total score and sub-scores except sleep disturbance, where CBT group had better effect (44). Two other studies (45, 46) on similar demographically comparable GAD subjects (n = 80 and 84) also supported the effectiveness of IPT in improving depression and anxiety measured by HAMA and HAMD, although CBT was superior in terms of improvement of quality of life. For social anxiety disorder patients, IPT and CBT performed better than the waiting list group in symptoms control (*p* < 0.05) (47); and patients received 12 weekly 120-min group IPT showed improvement in perceived self-efficacy in expressing positive affect (POS) and reduction in social avoidance and distress (*p* < 0.05) (48).

#### 3.2.7. PTSD or patients with childhood trauma

A study on adults affected by the Sichuan 2008 earthquake showed the efficacy of IPT for PTSD (49). Those who received IPT, compared with TAU, had a significantly greater reduction of PTSD diagnoses (51.9% versus 3.4% *p* < 0.01). Another study investigated 48 college students with history of childhood abuse. After randomization, 25 subjects received 12-session individual IPT (2 times per week, each session 30–40 min) while the rest had TAU (50). The result supported that IPT could alleviate the anxiety and depression symptoms and improve psychosocial functionality in college students with childhood trauma. The degree of improvement was also found to be positively related to alleviation of subjects’ psychological insecurity and enhancement of their resilience.

#### 3.2.8. Post-psychotic depression

PPD is the development of depression during the phase of remission of schizophrenia. Four studies found group and individual IPTs in addition to antidepressant can effectively alleviate depressive symptoms and improve overall functionality (51–54). Furthermore, it was also found that after treatment, the PANSS scores and WHO-DAS II scores of the combined group were significantly lower than the control group (*p* < 0.05), same as for compliance with antipsychotics (52, 54).

#### 3.2.9. Special population and other diagnoses

For young college students (age 18–19), Group IPT or CBT, comparing with no treatment, could significantly reduce the aggressiveness level and improve social support; and IPT was superior in terms of sub-scores of Impulsivity and Hostility of CC-BPAQ (55, 56). Although studies are still lacking in IPT use in other psychiatric diagnoses in Chinese population, one study investigated the use of IPT plus duloxetine in treatment of somatoform disorder patients. The result showed that individual IPT (2 times per week, 50–60 min per session, total 12 sessions) plus duloxetine had a better therapeutic effect (*p* < 0.05) on depression and anxiety symptoms comparing with duloxetine alone (57). Also included in the same study was an arm of CBT plus duloxetine. No significant difference was found between IPT and CBT groups in terms of HAMA and HAMD score reductions. As for bipolar depression, one study compared IPT-G (8 weekly 60-min sessions) plus Lithium (750 mg – 1,500 mg per day) and Lithium only. Both groups had high HAMD-17 (≥17) for depression and low YMRS (<20) for mania at entry point. The result showed IPT-G group not only was effective (*p* < 0.05) in reducing HAMD score for bipolar depression but also could improve the quality of life measured by WHOQOL_BREF (58).

Finally, Gao et al. has done extensive studies on effects of an IPT-oriented postnatal program for Chinese first-time mothers (59–61). The intervention consisted of a 60-min education session before hospital discharge plus one telephone follow-up within 2 weeks. The program utilized specific IPT techniques, such as information giving, use of affect, clarification, reviewing relationship and communication patterns, and providing social support. The results showed women receiving IPT-oriented education had significantly fewer depressive symptoms (*p* = 0.026), higher level of social support (*p* = 0.009) and better maternal role competence (*p* < 0.001) at 6 weeks postpartum as compared with those who received only routine postnatal care. Because of high adherence rate, they also concluded that the IPT-oriented childbirth education could be an acceptable treatment option to facilitate the Chinese first-time mothers’ transition to motherhood.

### 3.3. Meta-analysis

We performed a meta-analysis on 10 studies conducted on MDD subjects (22–31). Our qualitative analysis of the efficacy of IPT in this population highlighted two forest plots.

**Figure 2:**
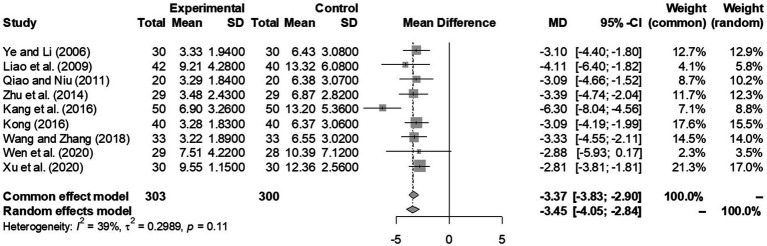
A forest plot outlining the overall HAMD score difference by 12 weeks in experimental (IPT + antidepressant) and control (antidepressant only) groups (*p* < 0.0001 from meta-analysis).

The first forest plot (Figure 2) included all 9 studies that compared HAMD scores between IPT plus medication and medication-only groups before treatment and at 12 weeks. The overall efficacy of IPT was analyzed using a random-effects model and yielded a significant score difference of −3.45 [−4.05; −2.84] between the two groups (*p* < 0.0001). This difference is in favor of IPT plus antidepressant group as lower HAMD score reflects better improvement in depression.

**Figure 3:**
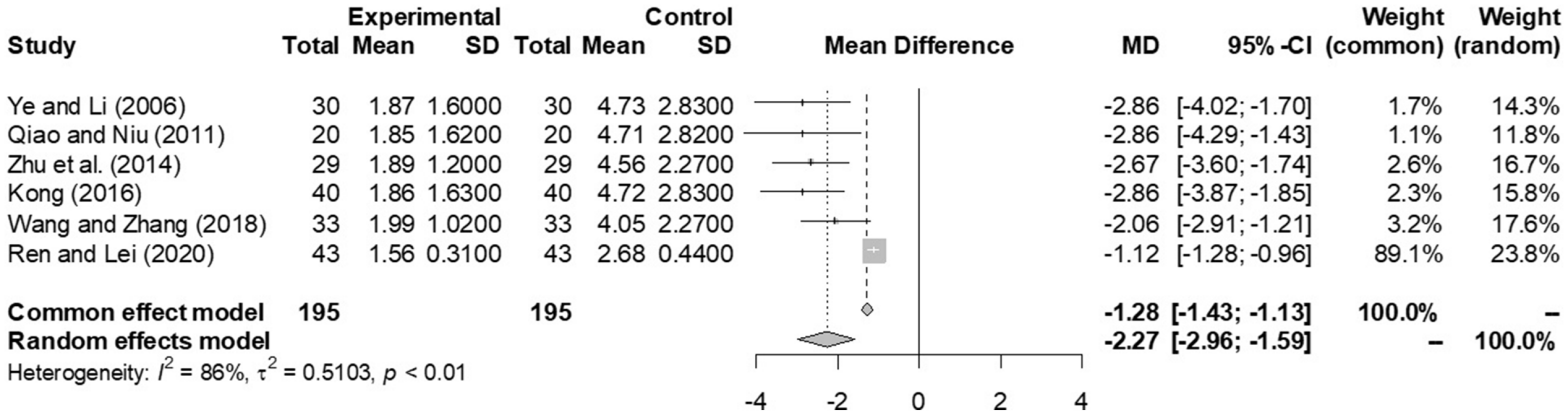
A forest plot outlining the overall SDSS score difference by 12 weeks in experimental (IPT + antidepressant) and control (antidepressant only) groups (*p* < 0.0001 from meta-analysis).

Including a study (29) that only used SDSS and SSRS as outcome measurement, a second forest plot (Figure 3) was generated to present the treatment efficacy of IPT by overall SDSS score change. One study only provided SDSS sub-scores (31) so was not included in this plot. Again, the random effects model resulted a score difference of −2.27 [−2.96; −1.59] in favor of the IPT group with a statistically significant difference (95% CI of −2.96 to −1.59, *p* < 0.001). Note that the random-effects model was the better approach since an I2 of 86%, the IPT outcome analysis revealed heterogeneity (*p* < 0.01) of all 6 studies included.

Unfortunately, due to limited number of studies, we were not able to conduct meta-analysis on IPT use in other Chinese populations.

### 3.4. Increasing number of IPT studies in China and their geographical distribution

**Figure 4:**
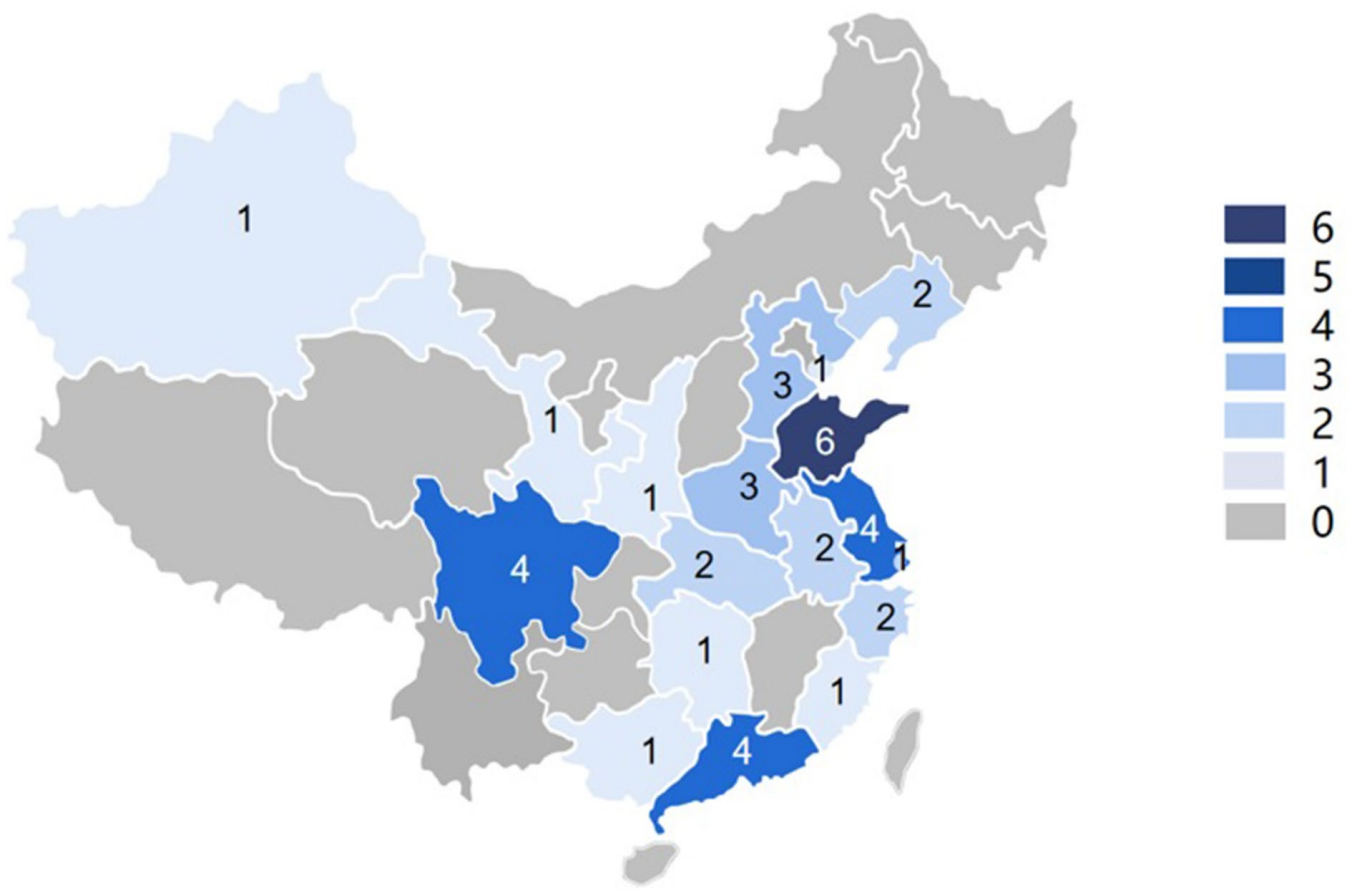
Map distribution of included IPT studies in mainland China.

Although IPT has not been commonly practiced in China, last two decades have seen an increasing number of clinical studies on IPT in Chinese population. While there were only 1 publication retrieved before 2006, we found 25 studies published in both Chinese and English journals after 2015. Figure 4 showed a geographical distribution map of IPT studies in mainland China. There was a noticeable regional difference in terms of the number of articles.

## 4. Discussion

We conducted a comprehensive review of IPT clinical studies in China. Forty relevant publications in Chinese and English were carefully reviewed and included in our analyses. For quality control reason, only randomized controlled trials were included. The findings of the reviewed studies and our meta-analysis showed IPT is an effective treatment for MDD, postpartum depression, GAD, post-psychotic depression, and some other mental health conditions. There were symptom improvements after the treatment and the differences were statistically significant pre- and post-, and comparing with the control groups. This is consistent with IPT studies conducted internationally throughout the past decades (1–13).

Out of 40 articles, 35 were published in Chinese and 5 were in English. All 35 Chinese articles were published in Chinese indexed academic journals. Majority (27/40, 67.50%) were 2–3 pages and about half (19/40, 47.50%) had less than 3,000 words in length. Due to the brevity and limited article length, some of the articles did not present enough details of study design, subject recruitment, and randomization process. Besides age, gender and psychiatric diagnoses, other baseline characteristics of participants were rarely described. Only 10 studies provided data on treatment adherence and attrition. An additional caveat is the uncertainty about the professional qualification of IPT therapists. The professional backgrounds of intervention providers varied from therapists and psychiatrists to nurses and midwives. The majority did not report any formal IPT training. Only 11 studies (27.5%) reported that providers undertook some professional training in IPT. One study reported that their therapist had IPT certificate. IPT supervision was also deficient. Only 10 studies (25%) mentioned the availability of supervision during the intervention. In addition, very few studies mentioned the need for and importance of cultural adaptation.

Nonetheless, in this systematic review, we carefully selected studies with research designs that are rigorous enough to rule out potential confounds and alternative explanations of treatment outcomes. In general, all studies included were RCTs and clearly described clinical settings, general study design, targeting psychiatric diagnosis, and IPT treatment details (individual vs. group, phases, frequency, and duration). Most of the studies used direct comparison between IPT and a control group which enabled us to clarify differences in treatment effects. All GAD studies included CBT as a known effective intervention for comparison. Moreover, the sample sizes of all studies were sufficient to draw statistical conclusions; and the outcome measures were standardized rating scales that have been validated in Chinese populations.

The results of the current study have several important implications for IPT research and knowledge dissemination in China. First, the findings of current studies have shown the efficacy of IPT in certain mental disorders particularly depression. It provides scientific evidence that IPT is effective and beneficial to Chinese population. Given the complex history of modern psychotherapy in China and its exponentially growing demand among nearly 1.4 billion citizens, our study paved the way for further promoting IPT as a treatment modality in mental health care, for developing evidence-based psychotherapy guidelines, and for improving the quality of clinical care. Second, the use of standardized measurement methods that allow for the assessment of treatment outcomes has become non-exceptional in psychotherapy studies in China. This is imperative in any mental health studies. Our study helps future Chinese IPT researchers understand the importance of this and hopefully can encourage those standard instruments to be used to supplement traditional clinical measures of treatment effectiveness, even in daily psychotherapy practices. Third, this review is a call for formal training and supervision of IPT therapists in China. This is important both in research and clinical practice. To maintain high standards and consistency in IPT, creation of a formal training and certification process in China has become a priority. This would not only help therapists improve their quality of care but also be recognized for their expertise in IPT. In addition, given that the majority of the trials were conducted in coastal and central part of China, we hope future IPT resources can be more properly allocated to cover those provinces and cities where mental health treatment was less accessible. Finally, incorporating cultural components into IPT practice is an imminent need. IPT was originally developed and tested in Western cultures. Questioning the extent of this treatment option’s effectiveness for Chinese people deep rooted with Confucius cultural background is legitimate. Even if interpersonal issues are universal, one important concern is whether IPT can be adapted to address Chinese cultures without changing its fundamental theories and elements. As none of the reviewed articles mentioned cultural modifications when practicing IPT in China, our study highlighted the importance of adapted and local IPT research and suggested for such a consideration in future related research. We emphasize this because the final acceptance of a Western psychotherapy model like IPT for people with a totally different cultural background depends on not only whether it is effective, but also how applicable it is in their values and beliefs.

Our study is not without limitations. First, the number of retrieved studies is relatively small, given a country with such a large population. Even for the most well-researched MDD population, there were only 10 randomized controlled trials included in the meta-analysis. Second, as mentioned above, some studies did not provide enough details on subject recruitment and participant demographics therefore the comparability and overall quality of the study may not be good enough to convince the efficacy of IPT as an intervention. Other issues included therapist qualification, subject selection bias, small samples, accuracy of diagnosis and concurrent use of other medications may be confounding factors that contributed significantly to the treatment outcomes. After all, none of the studies was conducted at more than one site. The results are not necessarily generalizable to other clinical sites or settings. Finally, like other meta-analyses, when we combined those studies using same diagnosis, outcome measures and same follow up schedules, the summary effect may ignore important differences between studies therefore be biased to certain extent.

## 5. Conclusion

In summary, in this comprehensive review of IPT use in China, we have identified the current best evidence for IPT efficacy in a variety of psychiatric disorders. Although the findings show the effectiveness of IPT in Chinese patients with certain psychiatric conditions, as this is the first systematic review of this topic, we caution not to overstate the apparent effects, given very few studies were available for each population. Still, due to lack of structured training and supervision opportunities, there remain substantial gaps in our knowledge of where, how, and when IPT should be implemented, and who can be trained and qualified to deliver IPT service in mainland China.

## Author contributions

WZ: conceptualization, methodology, validation, investigation, resources, writing – original draft and review and editing, and supervision. LT: conceptualization, methodology, data collection, reviewing, writing – original draft, and review and editing. FX and GY: methodology, data collection, reviewing, and writing – original draft. CL: statistical analysis and validation. SW: methodology, statistical analysis, validation, and writing – original draft. All authors contributed to the article and approved the submitted version.

## Funding

This study was funded by the Medical Science and Technology Project of Zhejiang Province, China (Funding Number: 2022503897).

## Conflict of interest

The authors declare that the research was conducted in the absence of any commercial or financial relationships that could be construed as a potential conflict of interest.

## Publisher’s note

All claims expressed in this article are solely those of the authors and do not necessarily represent those of their affiliated organizations, or those of the publisher, the editors and the reviewers. Any product that may be evaluated in this article, or claim that may be made by its manufacturer, is not guaranteed or endorsed by the publisher.
